# Symptoms, the GerdQ score and patients’ characteristics do not predict gastroesophageal reflux disease in patients with proton-pump-inhibitor-refractory reflux symptoms—results from a large prospective database

**DOI:** 10.7717/peerj.14802

**Published:** 2023-02-21

**Authors:** Joachim Labenz, Merlissa Menzel, Oliver Hirsch, Matthias Müller, Christian Labenz, Charles Christian Adarkwah

**Affiliations:** 1 Department of Medicine, Diakonie Hospital Jung Stilling, Siegen, Germany; 2 Phillips-Universität Marburg, Marburg, Germany; 3 FOM University of Applied Sciences, Siegen, Germany; 4 Department of Internal Medicine I, Johannes-Gutenberg Universität Mainz, Mainz, Germany; 5 Department of Heath Services Research, University of Maastricht, Maastricht, Netherlands; 6 Institute of General Practice, University of Marburg, Marburg, Germany

**Keywords:** GerdQ questionnaire, PPI therapy, Reflux disease, Persistent symptoms

## Abstract

**Background:**

The number of patients with proton pump inhibitor (PPI)-refractory reflux symptoms is underestimated since many patients resign after an unsuccessful therapy attempt. Thus, it would be useful having a non-invasive tool that can help identify true gastroesophageal reflux disease (GERD) patients in order to manage them early and properly. The GerdQ is a validated tool developed for this purpose but its applicability in PPI-refractory patients has not yet been investigated. Our aim was to investigate if reflux symptoms per se, the GerdQ and patients characteristics are suitable for non-invasive diagnosis of GERD in patients with PPI refractory reflux symptoms.

**Methods:**

A total of 500 patients from a prospectively recorded data base with PPI-refractory reflux symptoms were retrospectively analyzed. All patients received comprehensive diagnostic workup including EGD, pH-impedance measurement and manometry. GERD was diagnosed according to the recent Lyon consensus.

**Results:**

Of all patients enrolled in the study, 280 (56%) finally fulfilled the criteria for objectively verified GERD according to the Lyon consensus. There were no significant differences in age and gender between the patients with and without GERD, whereas the body mass index was significantly higher in the group with verified GERD, but the discriminative value was low (Welch-Test, *p* < .001, Cohen’s d = 0.39). Furthermore, there were no significant differences in the GerdQ values between the two groups. A GerdQ cutoff value ≥ 9 resulted in a sensitivity of 43% and specificity of 57% with a positive predictive value of 56% and a negative predictive value of 44%.

**Conclusion:**

Based on our study, neither symptoms and the GerdQ score nor patients’ characteristics are appropriate tools to distinguish between GERD and other causes for reflux symptoms in patients with PPI-refractory reflux symptoms.

## Introduction

Gastroesophageal reflux disease (GERD) affects about 20–25% of the adult population in the Western world (El-Serag et al., 2014; Antunes, Aleem & Curtis, 2021). Typical symptoms are heartburn and regurgitation. However, these are neither sensitive nor specific diagnostically on their own (Vakil et al., 2006). Symptom scores such as the Gastroesophageal Reflux Disease Questionnaire (GerdQ) have been developed and validated to improve symptom-based diagnosis in daily practice (Dent et al., 2010; Jonasson et al., 2013).

Typically, patients with reflux symptoms without presenting red flags and risk factors for complications are treated with proton pump inhibitors (PPI) (Katz, Gerson & Vela, 2013; Gyawali & Fass, 2018). However, 30–50% of patients remain symptomatic (Delshad et al., 2020; Labenz et al., 2016). In those situations, further clinical workup is indicated. As there is no gold standard for diagnosing GERD, a series of time-consuming and costly examinations is often necessary to establish or rule out the diagnosis, amongst other endoscopy with biopsy, impedance pH-metry, and high-resolution manometry (Spechler et al., 2019; Spechler, 2020). Given the large number of patients, this is not feasible with existing resources and represents a huge burden for health care systems. For this reason, a simple, noninvasive diagnostic tool is desirable to make the diagnosis of GERD likely in the presence of inadequate PPI response of reflux symptoms.

The aim of this study was to investigate if the GerdQ score is a suitable tool for symptom-based diagnosis of GERD in patients with PPI-refractory reflux symptoms. For this purpose, an exploratory analysis of prospectively collected data from the Reflux-Center Siegerland was performed.

## Methods

### Reflux Center Siegerland

The Reflux Center Siegerland was founded in 2014 in cooperation between two Siegen hospitals, to help patients with persistent reflux symptoms who are dissatisfied with their previous treatment. Based on comprehensive diagnostics at the Reflux Center the diagnoses are verified and conservative or operative options are discussed with the patients.

Before performing the diagnostic work-up within two working days, the patients needed to stop proton-pump-inhibitor (PPI) therapy, as far as still existing, at least for two weeks. By using specially tailored questionnaires, they were asked about their demographic characteristics, symptom frequency and strength, previous diagnostics and treatment (anamnestic and *via* medical report) as well as their treatment satisfaction. Furthermore, they were asked for their use of medication, pre-existing illness and previous *Helicobacter pylori* eradication. The demographic characteristics included name, gender, age, weight, height, smoking behavior and alcohol consumption. For the classification of the variety of symptoms, like heartburn, acid burp, regurgitation, epigastralgia, chest pain, nausea, sleep disturbance because of reflux, coughing and other atypical as well as individual symptoms, the patients got two charts for marking: one for the symptom severity in the last week (on a Likert scale from one to ten for every symptom) and the other for the symptom frequency (never, 1 day/week, 2–3 days/week, 4–7 days/week for every symptom).

In a next step, patients received comprehensive diagnostic workup, including esophagogastroduodenoscopy (EGD) with histological examination from the duodenum, stomach and esophagus, 24-hour pH-impedance measurement and high resolution manometry. Depending on symptoms some patients received additional tests, *e.g.*, esophageal swallow, if a motility disorder is suspected, or an octanoic acid test to exclude a gastric emptying disorder. Data collected during EGD included the presence of erosive esophagitis according to the Los Angeles Classification (Armstrong et al., 1996; Lundell et al., 1999), hernia size in centimeters, histologic abnormalities, and gastritis (categorized as A, B, C, depending on the histological findings). In case of Barrett mucosa, the extension was defined according to the Prague’s classification (Sharma et al., 2006). Based on pH impedance measurement, the following parameters were recorded: percent of time (%) spent with <pH 4 (acid exposure time of the esophagus) during day and night, DeMeester Score, symptom association probability (SAP), number of reflux events, and non-acid reflux. The high resolution manometry (HRM) was used to detect lower esophageal sphincter resting pressure and relaxation, presence of hernia (in cm), and esophageal motility disorder according to the Chicago classification (Bredenoord et al., 2012).

Based on the evaluated diagnostic results, the patients can be categorized as follows: Non erosive reflux disease (NERD) with proven acid reflux, hypersensitive esophagus without proven pathologic acid reflux but with significant correlation between physiological acid reflux and symptoms (SAP >95%), functional heartburn without any correlation between reflux events and symptoms, and erosive reflux disease (ERD), divided into mild ERD (Los Angeles A/B) or severe ERD (Los Angeles C/D).

Finally, according to the criteria of the Lyon consensus published in 2018 (Gyawali et al., 2018), the patients can be divided into those with GERD (functional heartburn excluded) and those without GERD.

### Definition of GERD

According to the Lyon consensus published in 2018, typical symptoms and response to therapy alone are not sufficient to diagnose GERD. Endoscopy results and pH or pH impedance measurement are required for a definitive diagnosis (Gyawali et al., 2018).

In accordance with the Lyon consensus, conclusive evidence for pathologic reflux are advanced erosive esophagitis (Los-Angeles grades C/D esophagitis), long segment Barrett‘s mucosa, peptic esophageal strictures or an acid exposure time (AET) >6% in the distal esophagus on pH or pH-impedance monitoring. In contrast, an acid exposure time <4% and <40 reflux episodes on pH-impedance measurement argue against pathologic reflux. According to the Lyon criteria, every conclusive finding is strong evidence for the presence of GERD. If the results are inconclusive or borderline, further findings from esophageal examinations (*e.g.*, biopsy and motor evaluation) can support the diagnosis (Gyawali et al., 2018).

In our study, the diagnosis of GERD was defined according to the criteria of the Lyon consensus (16) mentioned before. In the synopsis of the results collected from the diagnostic measurement, the patients were divided into those with GERD on the one hand and those without GERD on the other hand. GERD was diagnosed in the presence of erosive esophagitis Los Angeles C&D, an acid exposure time (AET) ≥ 6%, peptic esophageal strictures and/or long-segment Barrett‘s esophagus, whereas an AET <4% was considered as an exclusion criterion for GERD. In case of inconclusive or borderline results, such as an AET between 4–6% or erosive esophagitis Los Angeles A&B, the diagnosis was made by a synopsis of all findings in addition to expert opinion (interdisciplinary reflux board consisting of three experienced gastroenterologists and two visceral surgeons with a special focus on upper GI diseases).

### The GerdQ questionnaire (GerdQ)

The GerdQ is a validated non-invasive tool for diagnosing reflux disease in primary care. This questionnaire includes six items, four positive predictors (heartburn, regurgitation, sleep disturbance due to reflux-symptoms, use of over-the-counter antireflux medication), and two negative predictors (epigastric pain and nausea). Their frequency (0 day/week, 1 day/week, 2–3 days/week, 4–7 days/week) is specified on a Likert Scale (0–3) for the positive predictors and on a reversed Likert Scale (3–0) for the negative predictors (Jonasson et al., 2013; Jones et al., 2009). The GerdQ has its origin in the data of the large Diamond Study (4) and other questionnaires (Reflux Disease Questionnaire (RDQ) (Shaw et al., 2008), the Gastrointestinal Symptom Rating Scale (GSRS) (Revicki et al., 1998) and the Gastro-esophageal reflux disease Impact Scale (GIS) (Jones, Coyne & Wiklund, 2007)). Data of 308 primary care patients with upper abdominal symptoms completed the three questionnaires and the results were compared to those of endoscopy as well as 48-h Bravo-capsule pH monitoring. Using a cutoff of eight points in the GerdQ, the sensitivity was 64.6% and specificity was 71.4% (Jones et al., 2009). An initial diagnostic validation study of the GerdQ included 169 patients with suspected GERD. The results from the GerdQ questionnaire were compared to those of endoscopy, and in cases of unclear findings, also with pH metry. In this validation study, the sensitivity was 66% and the specificity was 64% using a cutoff of nine (Jonasson et al., 2013).

### Demographic characteristics of our study

In cooperation with the Reflux Center Siegerland, the characteristics, symptom manifestations, and diagnostic results of 500 PPI-refractory patients with and without GERD between 2014 and 2020 were collected. Of those, 243 (48.6%) patients were male and 257 (51.4%) are female, the age of the participants ranged from 15 to 89 years with a mean age of 52,84 (SD 15.1). The BMI varied from 16 kg/m^2^ as minimum to 44 kg/m^2^ as maximum with a mean BMI of 26,48 kg/m^2^ (SD 4.8). 362 of the participants (72.4%) were non-smokers, 71 (14.2%) were ex-smokers and 67 (13.4%) were smokers. Of all patients analyzed, 262 (52.4%) did not drink alcohol, whereas 177 (35.4%) consumed alcohol occasionally and 61 (12.2%) regularly.

Of the 500 patients included in our study, 457 (91.4%) participants already received previous diagnostic in form of an EGD before presenting to our Reflux Center the first time; only 8.2% received pH-metry in addition to the EGD. All participants have had an insufficient PPI treatment approach prior to their first visit at the Reflux Center.

At the Reflux Center Siegerland, all of the 500 included patients received EGD and, with only few exceptions, also pH-impedance measurement (*n* = 491) and high resolution manometry (*n* = 489). The study was performed according to the guidelines of the Declaration of Helsinki and approved by the Ethics Committee of the University of Essen Medical School (16-7125-BO). The data were retrospectively and anonymously analyzed. Hence, no informant consent of participants was needed according to the assessment of the ethics committee.

### Statistical analyses

Descriptive statistics with means and standard deviations for continuous variables and with percentages for categorical variables were calculated. Metric variables were tested regarding deviation from the normal distribution with the Shapiro–Wilk test and with tests regarding skewness und kurtosis.

Receiver operating characteristic (ROC) curves with 95% confidence intervals (CI) were used for estimating the optimal cutoff value to differentiate subjects with and without a diagnosis of GERD (Linden, 2006; Fawcett, 2006) and its associated sensitivity and specificity, positive and negative likelihood ratios (LR+ and LR-) and positive and negative predictive values (PPV and NPV). The Youden J index was used to find the optimal cut-off value as this index gives equal weight to sensitivity and specificity (Schisterman et al., 2005). A Youden J value of 0 indicates poor sensitivity and specificity for a specific cut-off score while a value of 1 indicates perfect sensitivity and specificity (Thompson et al., 2017).

Categorical variables were compared between the two groups using the Chi-square test with effect size Cramér-V. A Cramér-V ≥ .40 signals a large effect (Kotrlik & Williams, 2003). The Welch t test with effect size Cohen’s d was performed to compare means of two independent groups. The Welch t test is known to be robust against deviations from the normal distribution (Kubinger, Rasch & Moder, 2009). A Cohen’s d of 0.2 signals a small effect, a value of 0.5 a medium, and a value of ≥0.8 signals a large effect (Kotrlik & Williams, 2003). A two-sided *p*-value of .05 was considered to be statistically significant.

IBM SPSS 27, R version 4.02 and RStudio 1.2.5042 were used with R packages epiR, pROC (Robin et al., 2011), ROCit, lsr, QuantPsyc, and mosaic.

## Results

A total of 500 patients, who were consecutively evaluated in the Reflux Center, were analysed. Of these 500 patients, 280 patients (56%) fulfilled the criteria for objectively verified GERD (please see Methods section for further details). Sample characteristics are displayed in Table 1.

**Table 1:** Sample characteristics of subjects with and without diagnosis of Gastroesophageal reflux disease (GERD).

Variable	GERD	No GERD	Statistics
Gender	50% female	53.2% female	Chi-square *p* = .48, Cramér-V = .032
Age	53.9 (15.0)	51.5 (15.1)	Welch-Test, *p* = .08, Cohen’s *d* = − 0.16
BMI	27.3 (4.8)	25.5 (4.6)	Welch-Test, *p* < .001, Cohen’s *d* = 0.39

There were no significant differences regarding gender and age between subjects with and without GERD. Subjects with GERD had a significantly higher body mass index (BMI) than subjects without GERD. Hence, the effect size showed a small effect. The distribution of GerdQ raw scores in subjects with and without diagnosis of GERD is shown in Table 2. It can be seen that the distribution in each GerdQ score category is quite similar across the two groups.

**Table 2:** Distribution of GerdQ raw scores in subjects with and without diagnosis of gastroesophageal reflux disease (GERD).

			No GERD	GERD
GerdQscore	1		0	1
			0,0%	0,4%
	2		1	0
			0,5%	0,0%
	3		8	7
			3,6%	2,5%
	4		3	5
			1,4%	1,8%
	5		11	9
			5,0%	3,2%
	6		27	27
			12,3%	9,6%
	7		11	20
			5,0%	7,1%
	8		34	33
			15,5%	11,8%
	9		13	26
			5,9%	9,3%
	10		17	32
			7,7%	11,4%
	11		17	19
			7,7%	6,8%
	12		18	26
			8,2%	9,3%
	13		11	11
			5,0%	3,9%
	14		13	15
			5,9%	5,4%
	15		20	17
			9,1%	6,1%
	16		10	17
			4,5%	6,1%
	17		1	6
			0,5%	2,1%
	18		5	9
			2,3%	3,2%
Total			220	280
			100,0%	100,0%

Descriptive statistics of both groups regarding the GerdQ score are displayed in Table 3 which confirms the similiarity of both groups in the GerdQ score.

**Table 3:** Descriptive statistics of the GerdQ score in subjects with and without diagnosis of gastroesophageal reflux disease (GERD).

	Mean	SD	Min	Max	Shapiro–Wilk-Test	Skewness	Kurtosis
GERD	10.26	3.76	1	18	<.0001	*p* = .08	*p* = .01
No GERD	9.94	3.79	2	18	<.0001	*p* = .17	*p* = .005

**Figure 1:**
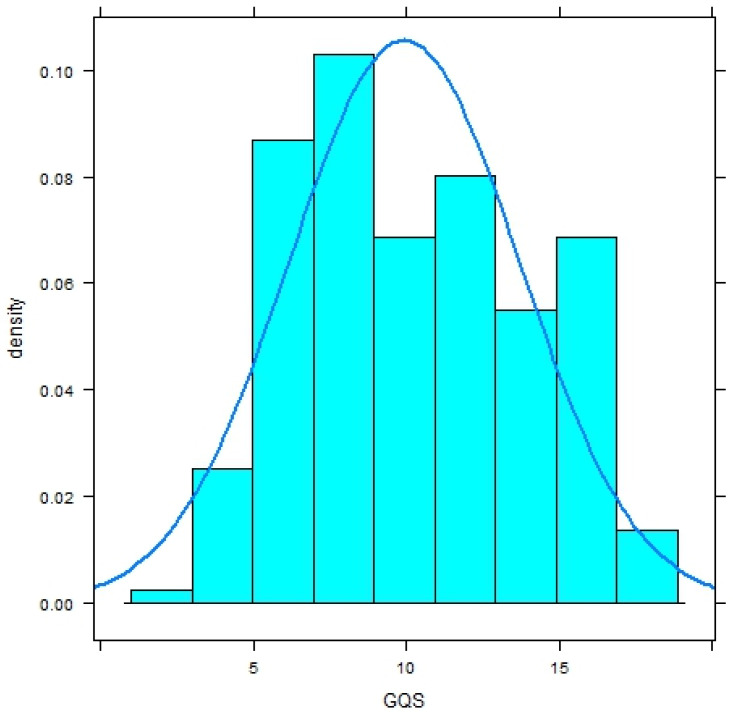
Histogram with normal curve of GerdQ scores in subjects without diagnosis of gastroesophageal reflux disease (GERD).

**Figure 2:**
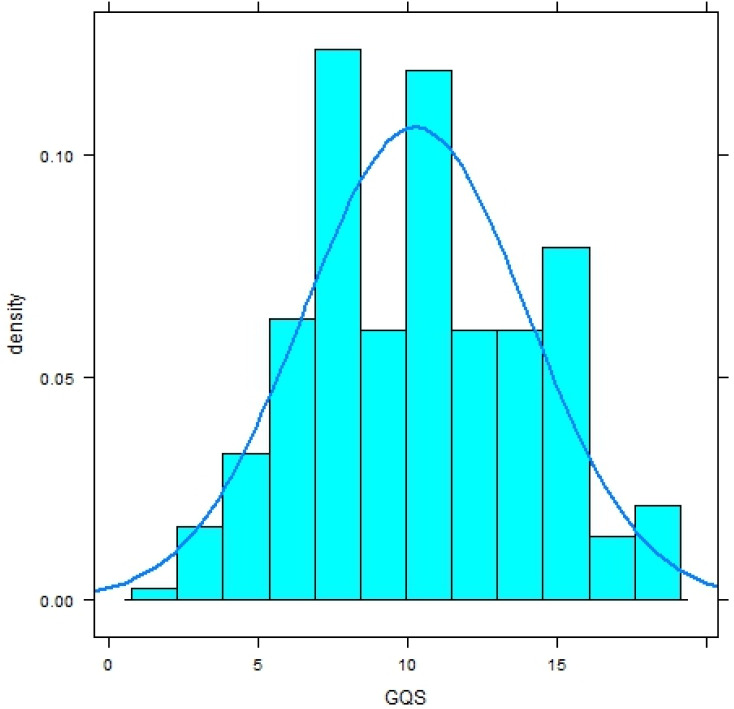
Histogram with normal curve of GerdQ scores in subjects with diagnosis of gastroesophageal reflux disease (GERD).

Both distributions significantly deviate from a normal distribution (Shapiro–Wilk-Test, *p* < .0001) and have a platykurtic shape (Figs. 1 and 2).

The Welch *t*-test resulted in a non-significant result (t(*df* = 468.91) = −0.94, p =.35) and Cohens’d signaled a negligible effect with *d* = 0.09.

**Figure 3:**
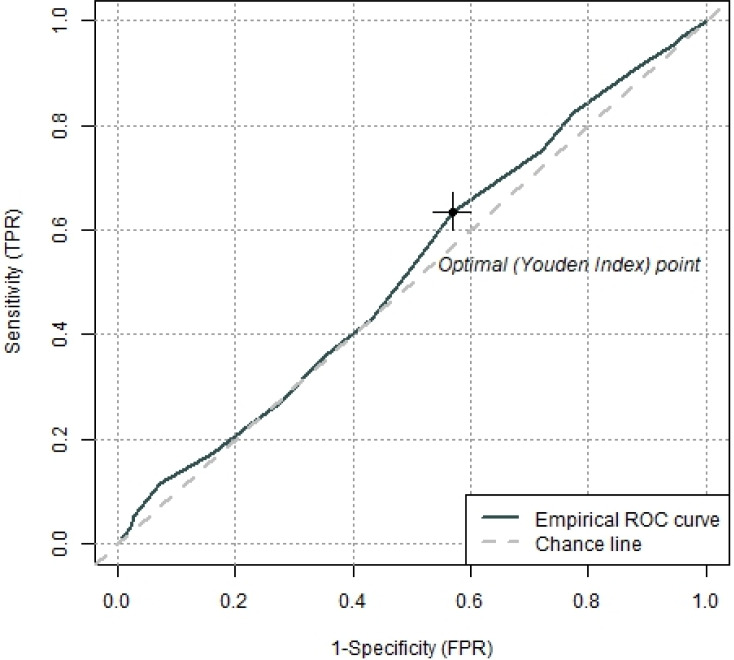
Receiver operating characteristics (ROC) curve with the optimal cut off value defined by the Youden index to separate subjects with and without diagnosis of GERD.

ROC analyses revealed that the GerdQ was not able to differentiate between the two groups. The area under the curve (AUC) was .525 (95% CI [.474–.576]), p =.34 which is slightly above chance level. The Youden Index (−.003, 95% CI [−.13–.12]) revealed a GerdQ score of 9 as an optimal cut-off value for the separation of subjects with and without diagnosis of GERD (Fig. 3) but it indicates poor sensitivity and specificity for this specific cut-off score.

Setting this threshold leads to the classification in Table 4 and the resulting parameters in Table 5.

In Table 4 a high false negative rate can be seen, giving a low sensitivity while specificity is higher with 57% but still far from being satisfactory. Positive and negative predictive values are also low and positive and negative likelihood ratios signal no ability of the test to differentiate between the two diagnostic groups (Table 5).

## Discussion

Our study showed for the first time that the GerdQ is not an appropriate tool to distinguish between GERD and other causes in patients with PPI-refractory reflux symptoms. Moreover, we could demonstrate in a large cohort with prospectively gathered data and an elaborated diagnostic work-up that about half of the patients complaining PPI-refractory reflux symptoms do not have true GERD based on the Lyon consensus (Gyawali et al., 2018).

Some studies in different countries provided evidence on the general applicability of the GerdQ, for example in Norway (Jonasson et al., 2013), Japan (Suzuki et al., 2013), Mexico (Zavala-Gonzales et al., 2014), China (Bai et al., 2013; Zhou et al., 2014; Wang et al., 2017), Korea (Gong et al., 2019), and in the Ukraine (Zaika et al., 2020). The number and characteristics of patients as well as the sensitivity and specificity of the GerdQ vary in the different studies. These studies basically were conducted in the primary care setting and—in contrast to our study—enrolled patients with reflux symptoms in general. Most of these studies show a positive correlation between the GerdQ score and the presence of reflux disease. For instance, another study by Lacy, Chehade & Crowell (2011) on 180 patients without PPI-therapy and 178 patients with PPI-therapy compared the results of 48h-wireless pH-monitoring to those of the GerdQ questionnaire. In contrast to the studies mentioned before, the results of Lacy et al. show both low sensitivity and specificity (Lacy, Chehade & Crowell, 2011). As mentioned already in the article of Vakil et al., 48h-wireless pH-metry as the only reference method for diagnosing GERD must be assessed critically as well as the fact that Lacy et al. evaluate the GerdQ partially on patients with an ongoing PPI therapy (Vakil & Kahrilas, 2012).

In contrast to previous studies we examined the GerdQ in patients with refractory reflux symptoms in the setting of a center. Furthermore, all of our study participants discontinued their PPI therapy at least two weeks before the study and the diagnosis of GERD was made according to the Lyon consensus criteria (Gyawali et al., 2018). Patients with refractory reflux symptoms represent an almost unexplored patient-population with a high burden of disease. For instance, when using a score as a diagnostic tool, it is very important to know to which patient group the score is applicable and to which it is not. In this context, it is important to know that, according to our study, the GerdQ cannot reliably predict GERD in patients with PPI-refractory reflux symptoms.

In addition, an important strength of our study is the large number of patients (*n* = 500) that were assessed in detail following a stringent evaluation plan. Furthermore, all patients discontinued PPI-therapy at least two weeks before presenting to our center for investigation, which is important for meaningful examination. Moreover, we used the combination of EGD, pH-impedance measurement and high resolution manometry as reference methods and new gold standard for the diagnosis of GERD according to modern standards (Lyon Consensus Gyawali et al., 2018).

**Table 4:** Classification table at threshold GerdQ score ≥ 9 for a diagnosis of gastroesophageal reflux disease (GERD).

	GERD	No GERD	
GerdQ score ≥9	120	95	215
GerdQ score <9	160	125	285
	280	220	

**Table 5:** Test evaluation parameters at a GerdQ score cut-off value ≥ 9.

	GERD	95% CI
Sensitivity	43%	37–49%
Specificity	57%	50–63%
Positive predictive value	56%	49–63%
Negative predictive value	44%	38–50%
Positive likelihood ratio	0.99	0.81–1.22
Negative likelihood ratio	1.01	0.86–1.17

## Conclusions

The data derived from our reflux center clearly showed that many patients suffering from so called reflux symptoms that are refractory to PPI therapy do not have reflux disease defined as troublesome symptoms as a result of reflux of stomach content into the esophagus (Vakil et al., 2006). We could clearly demonstrate that neither symptoms per se and the established GerdQ score nor patients characteristics are of any reasonable predictive value with respect to the confirmation or exclusion of GERD. These patients ultimately play a major role in primary care. Based on our findings patients with PPI-refractory reflux symptoms do need invasive diagnostic tests, especially those in whom antireflux surgery is considered.

In order to improve care of patients with persistent reflux symptoms despite PPI-therapy in the near future, the development of a stepwise approach is desirable to better distinguish between GERD and other diseases in this important patient population.

##  Supplemental Information

10.7717/peerj.14802/supp-1 Supplemental Information 1 Raw data

10.7717/peerj.14802/supp-2 Supplemental Information 2 Codebook

## Abbreviations

AET: acid exposure time
BMI: body mass index
CI: confidence intervals
EGD: esophagogastroduodenoscopy
ERD: erosive reflux disease
GerdQ: Gastroesophageal reflux disease questionnaire
GIS: Gastro-esophageal reflux disease Impact Scale
GERD: Gastroesophageal reflux disease
GSRS: Gastrointestinal Symptom Rating Scale
HRM: high resolution manometry
LR+: positive likelihood ratios
LR-: negative likelihood ratios
NERD: Non erosive reflux disease
NPV: negative predictive values
PPI: proton pump inhibitor
PPV: positive predictive values
RDQ: Reflux Disease Questionnaire
ROC: Receiver operating characteristic
SAP: symptom association probability

## Additional Information and Declarations

### Data Availability

The following information was supplied regarding data availability: The raw data are available as a Supplemental File.
