# Supplementary material for: Symptoms, the GerdQ score and patients’ characteristics do not predict gastroesophageal reflux disease in patients with proton-pump-inhibitor-refractory reflux symptoms—results from a large prospective database
Source: PeerJ. 2023 Feb 21;11:e14802. doi: 10.7717/peerj.14802 (PMC9951796; doi:10.7717/peerj.14802)
Supplement: Supplemental Information 2 [file peerj-11-14802-s002.docx]

**Codebook Dataset**

GES: Gender: 0 = male, 1 = female

ALT: Age: in years

BMI: BMI: in kg/m^2^

HIA: hernia (measured by endoscopy): 0-10 cm

HHN: hernia (measured by manometry): 0-10 cm

NERD: Non erosive reflux disease

0 = no

1 = yes, with pathological reflux

2 = yes, with normal reflux and pathological SAP(> 95%) = hypersensitive esophagus

3 = typical symptoms, but no pathological reflux and no SAP (= functional disorder)

SAP: symptom associated probability

ERD: erosive reflux disease

0 = no

1 = mild (Los Angeles A/B)

2 = severe (Los Angeles C/D)

BRT: Barrett`s esophagus

0 = no

1= short segment Barrett`s mucosa

2 = long segment Barrett`s mucosa

GERD: Gastro‐esophageal reflux disease 0 = not present, 1 = present

HIA_dich: hernia (measured by endoscopy): 0 = not present, 1 = present

HHN_dich: hernia (measured by manometry): 0 = not present, 1 = present

RGT_dich: regurgitation 0 = not present, 1 = present

NERD_123: NERD categories 1,2,3
